# Radioembolisation in patients with hepatocellular carcinoma that have previously received liver-directed therapies

**DOI:** 10.1007/s00259-018-3968-5

**Published:** 2018-03-07

**Authors:** Bruno Sangro, Carlo Ludovico Maini, Giuseppe Maria Ettorre, Roberto Cianni, Rita Golfieri, Daniele Gasparini, Samer Ezziddin, Philipp M. Paprottka, Francesco Fiore, Mark Van Buskirk, Jose Ignacio Bilbao, Rita Salvatori, Emanuela Giampalma, Onelio Geatti, Kai Wilhelm, Ralf Thorsten Hoffmann, Francesco Izzo, Mercedes Iñarrairaegui, Carlo Urigo, Alberta Cappelli, Alessandro Vit, Hojjat Ahmadzadehfar, Tobias Franz Jakobs, Rosa Sciuto, Giuseppe Pizzi, Secondo Lastoria, on behalf of the European Network on Radioembolization with Yttrium-90 resin microspheres (ENRY)

**Affiliations:** 1 Liver Unit, Clinica Universidad de Navarra, and Centro de Investigacion Biomedica en Red de Enfermedades Hepaticas y Digestivas (CIBEREHD), Avda. Pio XII, 36, 31008 Pamplona, Spain; 2 Nuclear Medicine, IFO Regina Elena National Cancer Institute, Rome, Italy; 3 General Surgery and Transplantation Unit, San Camillo Hospital, Rome, Italy; 4 Interventional Radiology, Ospedale S.M.Goretti, Latina, Italy; 5 Azienda Ospedaliero Universitaria di Bologna, Bologna, Italy; 6 Diagnostic and Interventional Radiology, Azienda Ospedaliera S. M. della Misericordia, Udine, Italy; 7 Nuclear Medicine, Universitätsklinik Bonn, Bonn, Germany; 8 Interventional Radiology, LMU Klinikum der Universität München, Munich, Germany; 9 Onco Interventional Radiology, Istituto Tumori Pascale, Naples, Italy; 10 Data Reduction LLC, 472 State Route 24, Chester, NJ USA; 11 Interventional Radiology, Clinica Universidad de Navarra, Pamplona, Spain; 12 Nuclear Medicine, Ospedale S.M.Goretti, Latina, Italy; 13 Nuclear Medicine, Azienda Ospedaliera S. M. della Misericordia, Udine, Italy; 14 Department of Radiology, University of Bonn, Bonn, Germany; 15 Hepatobiliary Surgery, Istituto Tumori Pascale, Naples, Italy; 16 Medical Oncology, S. Orsola-Malpighi Hospital, Bologna, Italy; 17 Interventional Radiology, IFO Regina Elena National Cancer Institute, Rome, Italy; 18 Nuclear Medicine, Istituto Tumori Pascale, Naples, Italy

**Keywords:** Radioembolisation, Yttrium-90 resin microspheres, Hepatocellular carcinoma, Selective internal radiation therapy

## Abstract

**Purpose:**

Radioembolisation is part of the multimodal treatment of hepatocellular carcinoma (HCC) at specialist liver centres. This study analysed the impact of prior treatment on tolerability and survival following radioembolisation.

**Methods:**

This was a retrospective analysis of 325 consecutive patients with a confirmed diagnosis of HCC, who received radioembolisation with yttrium-90 resin microspheres at eight European centres between September 2003 and December 2009. The decision to treat was based on the clinical judgement of multidisciplinary teams. Patients were followed from the date of radioembolisation to last contact or death and the nature and severity of all adverse events (AEs) recorded from medical records.

**Results:**

Most radioembolisation candidates were Child-Pugh class A (82.5%) with multinodular HCC (75.9%) invading both lobes (53.1%); 56.3% were advanced stage. Radioembolisation was used first-line in 57.5% of patients and second-line in 34.2%. Common prior procedures were transarterial (chemo)embolisation therapies (27.1%), surgical resection/transplantation (17.2%) and ablation (8.6%). There was no difference in AE incidence and severity between prior treatment subgroups. Median (95% confidence interval [CI]) survival following radioembolisation was similar between procedure-naive and prior treatment groups for Barcelona Clinic Liver Cancer (BCLC) stage A: 22.1 months (15.1–45.9) versus 30.9 months (19.6–46.8); *p =* 0.243); stage B: 18.4 months (11.2–19.4) versus 22.8 months (10.9–34.2); *p =* 0.815; and stage C: 8.8 months (7.1–10.8) versus 10.8 months (7.7–12.6); *p =* 0.976.

**Conclusions:**

Radioembolisation is a valuable treatment option for patients who relapse following surgical, ablative or vascular procedures and remain suitable candidates for this treatment.

## Introduction

Hepatocellular carcinoma (HCC) is the most common primary liver malignancy and a leading cause of cancer death worldwide [1, 2]. Curative treatment is feasible by resection or transplantation in early stage HCC (Barcelona Clinic Liver Cancer [BCLC] stage 0 or BCLC stage A) [1]. However, a high proportion of patients are not diagnosed until they are at an advanced stage (BCLC stage C) of the disease where curative interventions are not feasible. Tumour recurrence, or development of new tumour occurs in 70–80% of patients by 5 years after resection [3]. Percutaneous ablation, either radiofrequency ablation (RFA) or percutaneous ethanol injection, are options for BCLC stage 0-A HCC unsuitable for surgery. Locoregional therapy with transarterial chemoembolisation (TACE) is the preferred first-line treatment option for patients with intermediate stage (BCLC stage B) HCC [4, 5]. Sorafenib is an oral multi-tyrosine kinase and angiogenesis inhibitor that is the only approved systemic treatment for advanced stage (BCLC stage C) HCC, but while sorafenib has been shown to improve overall survival, its adverse event profile is an important limitation to its use [6, 7].

Radioembolisation—or selective internal radiation therapy (SIRT)—is a form of brachytherapy by the intra-arterial delivery of small (30–35 μm) biocompatible glass or resin microspheres loaded with the radionuclide yttrium-90 (^90^Y) to tumour sites in the liver. The ^90^Y microspheres lodge in the tumour capillaries plexus where they deliver a localised high-energy (0.93 MeV) dose of beta-radiation. Due to the limited tissue penetration of the beta-radiation, its tumouricidal activity is confined to the immediate proximity of the tumour, largely sparing normal liver parenchyma.

SIRT with ^90^Y microspheres, is used in specialist liver centres as part of the multimodal treatment of hepatocellular carcinoma (HCC) [8, 9]. However, the impact of prior treatment on tolerability and survival after radioembolisation has not been analysed.

## Methods

This study was a retrospective analysis of the data from the European Network on Radioembolisation with ^90^Y resin microspheres (ENRY) [8] to evaluate the impact of prior procedures on the safety and efficacy of radioembolisation in HCC. The patient population was identical to that reported in the original 2011 publication.

Consecutive patients with a confirmed diagnosis of HCC (either histologically proven or based on non-invasive European Association for the Study of the Liver criteria) and who had at least one follow-up visit after treatment were included in the analysis. Candidates for radioembolisation were considered from a population of patients who were not suitable for surgery (resection, liver transplantation), local ablation (percutaneous ethanol injection [PEI], radiofrequency ablation [RFA], cryoablation) or vascular (transarterial embolisation [TAE] or chemoembolisation [TACE]). Radioembolisation was performed with ^90^Y resin microspheres (SIR-Spheres; Sirtex Medical Limited, North Sydney, Australia). Patients may have received radioembolisation as a first-line therapy or after having progressed on previous surgical or non-surgical treatments (ablation, TAE or TACE). The decision to treat using radioembolisation was based on the clinical judgement of multidisciplinary teams at each centre. All patients provided informed consent for radioembolisation prior to treatment planning.

As part of a detailed pre-treatment work-up, patients underwent a thorough angiographic analysis and therapy simulation using ^99m^Tc-MAA to detect and occlude all collateral vessels that may carry microspheres to extrahepatic organs, and to rule out a high lung shunt, respectively. Depending upon the extent of tumour burden, patients were treated by either a segmental, lobar or whole-liver treatment approach. Further details of the treatment procedure are published elsewhere [10, 11].

Patients were followed from the date of radioembolisation to last contact or date of death. The nature and severity of all adverse events (AEs) were accessed from the medical records and graded using the Common Terminology Criteria for Adverse Events version 3.0 (CTCAE v3). An analysis of clinical and laboratory AEs was performed from baseline to the end of month 3 and the highest grade recorded from any one of the two analysis periods (from day 0 to 7 or from day 8 to month 3).

All statistical algorithms were conducted using SAS (Cary, North Carolina) analytical software. *P*-values for continuous variables were compared by one-way ANOVA, for nominal categorical variables by chi-square general association test, and for ordinal variables by Cochran-Mantel-Haenszel row mean scores. The change in CTCAE grade from baseline to 3 months (month 3 minus baseline) was calculated and compared among five subgroups by the Kruskal-Wallis test. Transitions in laboratory CTCAE grade 3–4 (yes/no) at month 0 to CTCAE grade 3–4 (yes/no) at month 3 was compared by the exact McNemar test. Kaplan-Meier analyses were used to estimate overall survivals with stratification by prior treatment group and by Barcelona Clinic Liver Cancer (BCLC) stage.

## Results

### Patient and treatment characteristics

Overall, 325 patients who were recruited from eight European centres in Germany, Italy and Spain between September 2003 and December 2009 were included in the analyses.

Candidates for radioembolisation were mostly cirrhotic (78.5%) and had a Child-Pugh class A (82.5%). Many had multinodular HCC (75.9%) invading both lobes (53.1%) and/or the portal vein (13.5% branch; 9.8% main), and had an altered Eastern Cooperative Oncology Group (ECOG) performance status (ECOG ≥1: 45.8%). According to the BCLC staging system, 56.3% of patients were classified as advanced (stage C).

**Fig. 1:**
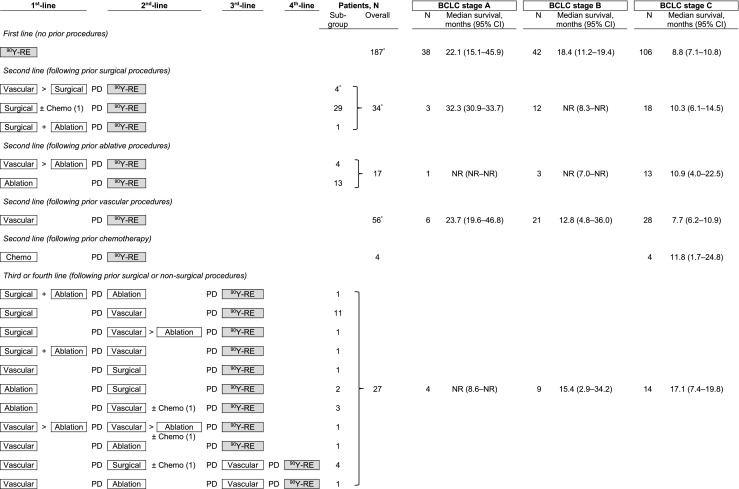
Distribution by prior procedure group and survival from day of first radioembolisation, stratified by BCLC stage. > denotes ‘followed by’

Just over half (187 patients; 57.5%) received radioembolisation as a first-line therapy, approximately one-third (111 patients; 34.2%) as second-line therapy and 27 patients (8.3%) as third or fourth-line treatment. Details of prior treatment and sequence are listed in Fig. 1 with baseline characteristics described in Table 1. The most common prior procedures overall were TACE or TAE in 88 patients (27.1%), followed by surgical resection or liver transplantation in 56 patients (17.2%) and ablation in 28 patients (8.6%).

**Table 1:** Baseline patient, disease and treatment characteristics by prior procedure (excluding chemotherapy)

Characteristic	Parameter	First-line (no prior procedure) (N = 187)	Second-line post-surgical (N = 34)	Second-line post-ablative (N = 17)	Second-line post-vascular (N = 56)	≥Third-line (N = 27)	*P* value across subgroups
Gender, *n* (%)	Male	154 (82.8)	25 (73.5)	13 (76.5)	48 (84.2)	22 (81.5)	0.650
	Female	33 (17.6)	9 (26.5)	4 (23.5)	8 (14.3)	5 (18.5)	
Age, years	Mean ± SD	64.7 ± 10.6	61.4 ± 11.6	69.7 ± 9.9	62.6 ± 11.7	68.1 ± 8.3	0.021
	Range	23–86	22–76	47–87	29–84	51–81	
ECOG	0	106 (57.0)	17 (50.0)	6 (35.3)	32 (57.1)	15 (55.6)	0.344
Performance	1	59 (31.7)	11 (32.4)	7 (41.2)	19 (33.9)	8 (29.6)	
Status, *n* (%) ^i^	2	20 (10.8)	5 (14.7)	4 (23.5)	4 (7.1)	4 (14.8)	
	3	1 (0.5)^a^	1 (2.9)	0	1 (1.8)	0	
Prior procedures, *n* (%)	Surgical (resection, transplant)	0	34 (100)	1 (5.9)	0	21 (77.8)	na
	Vascular (TACE/TAE)	0	4 (11.8)	4 (23.5)	57 (100)	24 (88.9)	na
	Ablation (PEI, RFA)	0	1 (2.9)	17 (100)	0	10 (37.0)	na
Cirrhosis, *n* (%)	Yes	150 (80.2)	18 (52.9)	16 (94.1)	46 (82.1)	22 (81.5)	0.002
Aetiology, *n* (%)	Hepatitis B	20 (10.7)	8 (23.5) ^a^	1 (5.9)	9 (16.4) ^a^	3 (11.1)	0.227
	Hepatitis C	79 (42.2)	12 (35.3)	11 (64.7)	28 (50.0)	12 (44.4)	0.284
Child-Pugh class, *n* (%)	A	153 (81.8)	30 (88.2)	11 (64.7)	46 (82.1)	24 (88.9)	0.264
	B	34 (18.2)	4 (11.8)	6 (35.3)	10 (17.9)	3 (11.1)	
Tumour burden (nodules), *n* (%)	1	56 (30.1)	5 (14.7)	2 (11.8)	10 (17.9)	5 (18.5)	0.382
	2–5	60 (32.3)	12 (35.3)	10 (58.8)	25 (44.6)	14 (51.9)	
	>5	70 (37.6)^a^	17 (50.0)	5 (29.4)	21 (37.5)	8 (29.6)	
Bilobar, *n* (%)	Yes	97 (51.9)	15 (45.5) ^a^	8 (47.1) ^a^	31 (55.4)	18 (66.7)	0.521
Extra-hepatic metastases, *n* (%)	Yes (lymph, bone, adrenal, pulmonary)	19 (10.2)	5 (14.7)	0	4 (7.1)	2 (7.4)	0.481
Portal vein occlusion, *n* (%)	Patent	138 (74.2)	31 (91.2)	11 (64.7)	43 (76.8)	23 (85.2)	0.432
	Branch	29 (15.6)	1 (2.9)	3 (17.6)	8 (14.3)	3 (11.1)	
	Main	19 (10.2)	2 (5.9)	3 (17.6)	5 (8.9)	1 (3.7)	
Ascites, *n* (%)	Yes	22 (13.8) ^i^	5 (16.1) ^c^	1 (5.9)	5 (9.4) ^c^	3 (11.5) ^a^	0.782
Encephalopathy, *n* (%)	Yes	5 (3.1) ^i^	0 ^c^	0	2 (3.8) ^c^	0 ^a^	0.635
BCLC stage, *n* (%)	A	38 (20.3)	3 (8.8)	1 (5.9)	6 (10.7)	4 (14.8)	0.477
	B	42 (22.5)	12 (35.3)	3 (17.6)	21 (37.5)	9 (33.3)	
	C	106 (56.7)	18 (52.9)	13 (76.5)	28 (50.0)	14 (51.9)	
	D	1 (0.5)	1 (2.9)	0	1 (1.8)	0	
Alfa-fetoprotein	>400 ng/mL, n (%)	62 (35.4) ^h^	10 (29.4)	7 (43.8) ^a^	20 (35.7)	9 (33.3)	0.901
Total bilirubin	Mean ± SD, mg/dL	1.2 ± 0.63 ^a^	0.8 ± 0. 38	1.2 ± 0.50 ^a^	1.0 ± 0.53	0.9 ± 0.40	0.003
	>1.5 mg/dL, n (%)	37 (19.9) ^a^	2 (5.9)	3 (18.8) ^a^	9 (16.1)	2 (7.4)	0.202
Albumin	Mean ± SD, g/dL	3.6 ± 0.65 ^h^	4.1 ± 2.48 ^f^	3.3 ± 0.69 ^c^	3.6 ± 0.57 ^e^	3.5 ± 0.87 ^b^	0.116
	<3.5 g/dL, n (%)	69 (39.4) ^h^	11 (40.7) ^f^	9 (64.3) ^c^	21 (41.2) ^e^	13 (52.0) ^b^	0.359
INR	Mean ± SD	1.2 ± 0.25 ^a^	1.0 ± 0.20 ^b^	1.2 ± 0.32 ^a^	1.2 ± 0.19 ^a^	1.2 ± 0.32	0.049
	>1.2, n (%)	47 (25.3) ^a^	4 (12.5) ^b^	5 (31.3) ^a^	13 (23.2)	6 (22.2)	0.556
ALT	Mean ± SD, U/L	63.6 ± 50.9 ^d^	61.6 ± 54.07	65.1 ± 40.1 ^a^	54.8 ± 51.0	56.2 ± 29.1	0.778
Creatinine	Mean ± SD, mg/dL	0.9 ± 0.29 ^g^	0.9 ± 0.30	0.9 ± 0.21	1.0 ± 0.50 ^b^	0.9 ± 0. 29	0.872
Occlusion of non-target arteries, *n* (%)		110 (58.8)	29 (85.3)	12 (70.6)	26 (46.4)	17 (63.0)	0.006
Activity administered	Median, GBq	1.7	1.5	1.3	1.5	1.6	0.018
	Range	0.3–4.0	0.6–2.5	0.5–2.0	0.3–3.4	0.8–2.6	
Target treatment, *n* (%)	Whole Liver	76 (40.6)	26 (76.5)	9 (52.9)	24 (42.9)	19 (70.4)	0.010
	Right Lobe	78 (41.7)	4 (11.8)	7 (41.2)	22 (39.3)	6 (22.2)	
	Left Lobe	17 (9.1)	3 (8.8)	1 (5.9)	7 (12.5)	0	
	Segmental	16 (8.6)	1 (2.9)	0	3 (5.4)	2 (7.4)	
Target tumour volume, mL	Median	250.0	165.2	256.0	170.9	115.4	0.461
	Range	2.2–1908	3.0–3326	3.0–1932	12.0–4000	8.0–1300	
Target liver volume, mL	Median	1395	1483	1441	1206	1533	0.972
	Range	98–3816	115–4826	256–3572	103–5566	240–2807	
Target tumour burden, %	Median	24.7	14.3	17.9	13.6	10.7	0.110
	Range	0.1–100	0.2–100	0.5–100	1.1–100	1.1–91.6	
Whole tumour burden, %	Median	14.3	9.1	17.9	11.8	8.5	0.297
	Range	0.1–75.0	0.2–68.9	0.3–54.1	1.0–71.9	0.5–53.7	
Number of treatments, *n* (%)	1	171 (91.4)	31 (91.2)	17 (100)	54 (96.4)	23 (85.2)	0.215
	2	15 (8.0)	2 (5.9)	0	2 (3.6)	3 (11.1)	
	3	1 (0.5)	1 (2.9)	0	0	1 (3.7)	

*ALT* alanine transaminase, *BCLC* Barcelona Clinic Liver Cancer, *PEI* percutaneous ethanol injection, *RFA* radiofrequency ablation, *TAE* transarterial embolisation, *TACE* transarterial chemoembolisation, *na* not applicable

*p-*value for continuous variables by one-way ANOVA, and *p*-value for nominal categorical variables by chi-square general association test, and ordinal variables (e.g. ECOG) by Cochran-Mantel-Haenszel row mean scores. Percentages calculated on available data; n (%) unless stated; sites of extrahepatic metastases included mainly lymph nodes but also bone, adrenal and lung

Table excludes four patients (all BCLC stage C) who received radioembolisation at second-line following prior chemotherapy; missing baseline data on ^a^One patient ^b^Two patients ^c^Three patients ^d^Four patients ^e^Five patients ^f^Seven patients ^g^Eight patients ^h^12 patients ^i^27 patients

As shown in Table 1, baseline characteristics varied little between the no-prior procedure and any-prior procedure groups, with a similar ECOG performance status and BCLC stage across all groups. There were, however, a few notable between- and intra-group differences. The incidence of cirrhosis at baseline was lower among second-line post-surgical patients than in the other subgroups (*p =* 0.002) and mean baseline total bilirubin and INR were lowest in the post-surgical group (*p =* 0.003 and 0.049, respectively). Not surprisingly, treatment was most likely to be delivered as a whole-liver (or whole-remnant) procedure in second-line post-surgical patients and in those treated at third-line or beyond (*p* = 0.010), and occlusion of the non-target arteries prior to administration was more likely to be performed in the second-line post-surgical cohort (*p =* 0.006). A significantly higher activity was administered to treatment-naïve patients (median 1.7 versus 1.5 GBq; *p* = 0.003).

Patients that had undergone a prior procedure tended to have a smaller tumour burden (14.1% versus 11.2%; *p =* 0.060), were less likely to have an elevated baseline total bilirubin compared with patients who were procedure-naïve (mean ± SD: 1.0 ± 0.48 versus 1.2 ± 0.63 mg/dL; *p* < 0.001), and were less likely to receive a whole-liver treatment than a lobar/segmental approach (whole-liver: 40.8% versus 58.2%, respectively; *p =* 0.017).

Patients with any prior surgical procedure were less likely to have an elevated total bilirubin (mean ± SD: 0.8 ± 0.38 versus 1.1 ± 0.52; *p =* 0.014), cirrhosis (64.3% versus 84.6%; *p =* 0.008), portal vein occlusion (8.9% versus 26.9%; *p =* 0.035) or hepatitis C aetiology (35.7% versus 55.1%; *p =* 0.035) at baseline compared with those receiving other prior procedures. Nearly three-quarters (73.2%) of these post-surgical patients received whole-liver radioembolisation of the remnant liver (a statistically higher proportion than in the remaining cohorts [47.4%]; *p =* 0.030). By contrast, patients with any prior ablation were older (mean ± SD: 68.5 ± 9.9 versus 63.2 ± 11.3 years; *p =* 0.025), and appeared more likely to have cirrhosis (89.7% versus 72.6%; *p =* 0.082), compared to those receiving other prior procedures. Finally, patients who had had any prior TAE or TACE were less likely to have non-target arteries occluded (54.5% versus 78.3%; *p =* 0.008) compared with those receiving other prior procedures.

### Safety and tolerability

There were no significant differences between subgroups according to prior procedures in the reporting of clinical AEs (Table 2) or changes in CTCAE grade from baseline to 3 months (Table 3). The incidence of commonly reported mild-to-moderate procedure-related events in the first 7 days after radioembolisation (including fatigue, nausea, and vomiting, pain and fever) was numerically higher in the prior-ablative subgroup but not statistically different from the remainder of patients. This is likely to be linked to a higher, but not significantly different, proportion of patients with more advanced, symptomatic disease reflected by higher rates of ECOG performance status ≥1, tumour burden, cirrhosis, and Child B class (Table 1). In this setting, the prior ablative subgroup, not surprisingly, also had higher (but not significantly different) pre- and post-radioembolisation grades of total bilirubin, albumin, ALT, INR and platelets, although there did not appear to be any additional increase in post-treatment grades in this subgroup as a consequence of the existing dysfunction (Table 1).

**Table 2:** Main procedure-related clinical adverse events to 3 months post-treatment by severity (CTCAE v3)

CTCAE	Radioembolisation subgroup	Number of patients	CTCAE v3: Number (%) of patients			*P* value across subgroups
			All Grades	Grade 3	Grade 4/5	
Fatigue	1st-line	187	91 (48.7)	5 (2.7)	0	0.108
	2nd-line post-surgical	34	22 (64.7)	0	0	
	2nd-line post-ablative	17	14 (82.4)	0	0	
	2nd-line post-vascular	56	30 (53.6)	2 (3.6)	0	
	≥3rd-line	27	19 (70.4)	2 (7.4)	0	
Nausea and/or vomiting	1st-line	187	59 (31.6)	0	0	0.435
	2nd-line post-surgical	34	11 (32.4)	0	0	
	2nd-line post-ablative	17	8 (47.1)	0	0	
	2nd-line post-vascular	56	16 (28.6)	0	0	
	≥3rd-line	27	10 (37.0)	1 (3.7)	0	
Abdominal pain	1st-line	187	40 (21.4)	3 (1.6)	0	0.240
	2nd-line post-surgical	34	11 (32.4)	1 (2.9)	0	
	2nd-line post-ablative	17	8 (47.1)	0	0	
	2nd-line post-vascular	56	18 (32.1)	0	0	
	≥3rd-line	27	10 (27.0)	1 (3.7)	0	
Fever	1st-line	187	18 (9.6)	0	0	0.173
	2nd-line post-surgical	34	7 (20.6)	0	0	
	2nd-line post-ablative	17	5 (29.4)	0	0	
	2nd-line post-vascular	56	6 (10.7)	0	0	
	≥3rd-line	27	4 (14.8)	0	0	
GI ulceration	1st-line	187	5 (2.7)	2 (1.1)	0	0.353
	2nd-line post-surgical	34	1 (2.9)	1 (2.9)	0	
	2nd-line post-ablative	17	1 (5.9)	0	0	
	2nd-line post-vascular	56	4 (7.1)	2 (3.6)	1 (1.8)	
	≥3rd-line	27	1 (3.7)	0	0	

*CTCAE v3* Common Terminology Criteria for Adverse Events version 3.0, *GI* gastrointestinal

Procedure-related events (fatigue, nausea and vomiting, abdominal pain and fever) were evaluated from day 1 to day 7. Radiation-related events (long-term fatigue and GI ulceration) were evaluated from day 8 to month 3. The highest grade of adverse event reported by each patient within each time interval is reported; *p* value for CTCAE distribution comparison between cohorts by Cochran-Mantel-Haenszel row mean score test statistic

**Table 3:** Comparison of laboratory adverse events by severity (CTCAE v3) between baseline and month 3

CTCAE	Radioembolisation sub-group	Number of patients	Assessment visit				*P* value across sub-groups ^†^
			Pre-radioembolisation %		Month 3%		
			All grade	Grade ≥ 3	All grades	Grade ≥ 3	
Total bilirubin	1st-line	167	26.3	0	53.9	4.8	0.628
	2nd-line post-surgical	34	5.9	0	32.4	5.9	
	2nd-line post-ablative	16	37.5	0	62.5	6.3	
	2nd-line post-vascular	48	20.8	0	47.9	10.4	
	≥3rd-line	24	12.5	0	33.3	4.2	
Albumin	1st-line	137	37.2	0	38.7	1.5	0.931
	2nd-line post-surgical	26	30.8	0	30.8	0	
	2nd-line post-ablative	13	61.5	0	69.2	0	
	2nd-line post-vascular	39	38.5	0	38.5	0	
	≥3rd-line	19	36.8	0	42.1	0	
ALT	1st-line	151	62.9	2.6	60.3	4.6	0.705
	2nd-line post-surgical	33	45.5	3.0	60.6	0	
	2nd-line post-ablative	15	73.3	0	66.7	6.7	
	2nd-line post-vascular	46	45.7	0	41.3	0	
	≥3rd-line	24	75.0	0	62.5	4.2	
INR	1st-line	162	23.5	0	30.9	1.9	0.889
	2nd-line post-surgical	31	12.9	0	29.0	0	
	2nd-line post-ablative	14	28.6	0	42.9	0	
	2nd-line post-vascular	43	25.6	0	34.9	4.7	
	≥3rd-line	24	20.8	0	29.2	0	
Creatinine	1st-line	157	8.3	0	10.8	1.3	0.344
	2nd-line post-surgical	33	6.1	0	3.0	0	
	2nd-line post-ablative	15	6.7	0	13.3	0	
	2nd-line post-vascular	45	11.1	2.2	20.0	4.4	
	≥3rd-line	23	8.7	0	13.0	0	
Platelets	1st-line	156	42.3	1.9	54.5	1.9	0.294
	2nd-line post-surgical	29	17.2	0	24.1	3.4	
	2nd-line post-ablative	14	78.6	7.1	78.6	0	
	2nd-line post-vascular	44	56.8	4.5	59.1	6.8	
	≥3rd-line	22	50.0	0	50.0	4.5	

*ALT* alanine transaminase, *CTCAE* Common Terminology Criteria for Adverse Events version 3.0, *INR* International Normalised Ratio, *nr* not reported

All events were evaluated from baseline to month 3^†^ The change in CTCAE grade from baseline to 3 months (month 3 minus baseline) is compared between sub-groups by the Kruskal-Wallis test. Differences in laboratory values between baseline and month 3 were also assessed by McNemar test regarding Grade 3–4 CTCAE (Yes/No) at month 3 versus Grade 3–4 CTCAE at month 0 and were statistically significant (*p* < .05) for total bilirubin in the overall cohort (data not shown; *p* < 0.001), in the 1st-line cohort (*p* = 0.008), and a trend in 2nd-line post-vascular cohort (*p =* 0.063)

### Overall survival

**Fig. 2:**
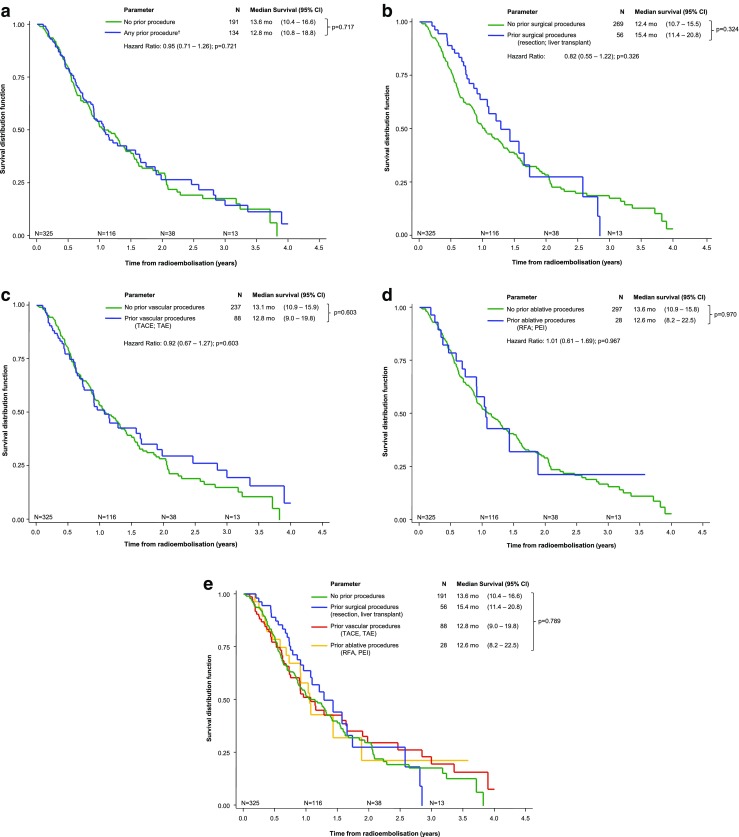
Kaplan-Meier survival analysis of patients with unresectable hepatocellular carcinoma treated with ^90^Y–resin microspheres stratified by prior procedure history. **a** Any prior procedure (surgical, vascular and/or ablation). **b** Prior surgical procedure (resection; transplantation). *c* Prior vascular procedure (TACE; TAE). **d** Prior ablative procedure (RFA; PEI). **e** Composite of prior procedures or none

Overall survival following radioembolization was similar in the procedure-naive group to those who had received any prior procedure (Fig. 2a). Similarly, stratification of patients by BCLC stage showed that median (95% CI) survivals were not statistically different in the procedure-naive group and those who received radioembolisation second line in patients with BCLC stage A: 22.1 months (15.1–45.9) versus 30.9 months (19.6–46.8; *p =* 0.243); BCLC stage B: 18.4 months (11.2–19.4) versus 22.8 months (10.9–34.2, *p =* 0.815); or BCLC stage C: 8.8 months (7.1–10.8) versus 10.8 months (7.7–12.6, *p =* 0.976). Furthermore, stratifying patients by prior procedure, whether surgical (Fig. 2b), vascular (Fig. 2c), or ablative (Fig. 2d) showed that survival differed little by prior treatment type.

## Discussion

Our analysis of 325 patients showed that patients with HCC who have failed prior procedures can be treated effectively with radioembolisation and have outcomes similar to those who had no prior procedures. This is perhaps analogous to the observation that patients with progression or intolerance to sorafenib had a better-than-expected outcome when treated with systemic agents, showing that later lines of therapy can still be effective after earlier lines of treatment have failed [12, 13]. These data may be indicative of the tumours in patients with good functional reserve, who are eligible for additional treatment, having a less aggressive nature upon progression.

It is quite reassuring to observe that prior treatment did not significantly increase the rate of severe AEs post-radioembolisation, particularly after resection where liver functional reserve is diminished, and following vascular procedures where the liver vascular network is altered. Minor differences in adverse event rates post-radioembolisation (e.g. fatigue and abdominal pain) tended to reflect existing dysfunction recorded at baseline. Survival was particularly promising for a small subset of patients (34/325 patients; 10.5% of the overall cohort) with early or intermediate-stage disease who received radioembolisation after failing prior surgery (with or without TACE or embolisation). These data suggest that the hypothesis that radioembolisation may be an alternative to TACE earlier in the treatment paradigm is worth further study. Due to the small patient numbers, we were unable to draw any conclusions on the safety or efficacy of radioembolisation in patients who had received prior systemic therapy.

Often the sequential use of locoregional therapies is key in optimising outcomes for patients with localised disease. The nature and duration of prior treatment often determines subsequent treatment options. There are, however, few published data on the relative value of conducting TACE before radioembolisation, or vice versa. Our study has shown that in patients who are eligible for radioembolisation, prior failure of TACE does not affect the subsequent outcomes of treatment using ^90^Y resin microspheres. However, we emphasise that patients who are suitable candidates for radioembolisation after TACE are necessarily expected to have a more favourable prognosis (i.e., no or less extrahepatic progression, and less rapid progression) than patients who are unsuitable for radioembolization after TACE. With the availability of effective alternatives to conventional TACE, an individualised approach to treatment is needed that is appropriate both to the intention of treatment (i.e. palliation or down-sizing) and the patient’s health status. Such decisions should be based on the collective experiences of the multidisciplinary team.

While there is evidence to suggest that repeating TACE prolongs survival, it can be a difficult decision to know when to stop TACE and consider alternative treatment. In the opinion of some experts, patients should receive no more than two sessions of TACE in the presence of persistent tumoural activity on imaging [14, 15]. This is supported by two recent papers from researchers in Vienna, who used Assessment for Retreatment with TACE (ART) scoring to show that patients with deteriorating liver function, defined by rising aspartate aminotransferase (AST) and Child-Pugh score in the absence of radiologic tumour response, are poor candidates for ongoing TACE [16, 17]. Furthermore, although SIRT is an effective salvage therapy in patients after TACE, the feasibility of SIRT tends to decrease in patients who have undergone more than four previous TACE sessions [18]. Finally, better tumour control has been documented with SIRT, albeit with ^90^Y glass microspheres (TheraSphere®; MDS Nordion, Ottawa, ON, Canada), when compared with conventional TACE in a randomised prospective trial [19]. This study showed a significantly longer median time to progression (>26 months) in the SIRT group than in the TACE group (6.8 months; *p* = 0.0012). Such evidence may strengthen the argument for the potential use of SIRT earlier in the disease course.

Personalised dosimetry was not conducted in this study, nor was it performed in either of the large randomised controlled trials on the use of SIRT in locally advanced HCC [20, 21]. However, two earlier trials have indicated that a delivered dose calculated based on ^99m^Tc-MAA quantification may affect treatment outcome. A study with ^90^Y resin microspheres reported that survival was significantly better in patients whose tumours received >120 Gy (median survival 55.9 weeks) than those who received lower doses (median survival 26.2 weeks; *p* = 0.005) [22]. In another study with ^90^Y glass microspheres, median OS was 4.4 months in patients whose dose was <205 Gy versus 15.7 months in patients whose dose was ≥205 Gy (*p* = 0.0004) [23]. These studies indicate a that personalised dosimetry according to the finding of ^99m^Tc-MAA angiography may optimise the outcome of SIRT. When individualised dosimetry is used in clinical practice, the benefit of SIRT may be greater than that seen in the large randomised controlled trials.

On the whole, survival following radioembolisation was not statistically significantly different between treatment naïve patients and patients who had received at least one prior procedure (13.6 months and 12.8 months). These data are strikingly similar to the results from the SHARP study, which found that median overall survival with sorafenib was 11.9 months in both subgroups of patients who had received prior curative treatment (resection or ablation) or TACE [24]. In conclusion, this analysis shows that radioembolisation is a valuable treatment option for patients who relapse following surgical, ablative or vascular procedures and who remain suitable candidates for this treatment.

## References

[1] Crissien AM, Frenette C (2014). Current management of hepatocellular carcinoma. Gastroenterol Hepatol (N Y)..

[2] Balogh J, Victor D, Asham EH, Burroughs SG, Boktour M, Saharia A (2016). Hepatocellular carcinoma: a review. J Hepatocell Carcinoma.

[3] Kwon SK, Yun SS, Kim HJ, Lee DS (2014). The risk factors of early recurrence after hepatectomy in hepatocellular carcinoma. Ann Surg Treat Res.

[4] European Association for Study of Liver; European Organisation for Research and Treatment of Cancer (2012). EASL-EORTC clinical practice guidelines: management of hepatocellular carcinoma. Eur J Cancer.

[5] Bruix J, Sherman M (2011). American Association for the Study of Liver Diseases. Management of hepatocellular carcinoma: an update. Hepatology.

[6] Llovet JM, Ricci S, Mazzaferro V, Hilgard P, Gane E, Blanc JF (2008). Sorafenib in advanced hepatocellular carcinoma. N Engl J Med.

[7] Cheng AL, Kang YK, Chen Z, Tsao CJ, Qin S, Kim JS (2009). Efficacy and safety of sorafenib in patients in the Asia-Pacific region with advanced hepatocellular carcinoma: a phase III randomised, double-blind, placebo-controlled trial. Lancet Oncol..

[8] Sangro B, Carpanese L, Cianni R, Golfieri R, Gasparini D, Ezziddin S (2011). Survival after yttrium-90 resin microsphere radioembolization of hepatocellular carcinoma across Barcelona clinic liver cancer stages: a European evaluation. Hepatology.

[9] Salem R, Lewandowski RJ, Mulcahy MF, Riaz A, Ryu RK, Ibrahim S (2010). Radioembolization for hepatocellular carcinoma using Yttrium-90 microspheres: a comprehensive report of long-term outcomes. Gastroenterology.

[10] Kennedy A, Coldwell D, Sangro B, Wasan H, Salem R (2012). Radioembolization for the treatment of liver tumors general principles. Am J Clin Oncol.

[11] Gil-Alzugaray B, Chopitea A, Inarrairaegui M, Bilbao JI, Rodriguez-Fraile M, Rodriguez J (2013). Prognostic factors and prevention of radioembolization-induced liver disease. Hepatology.

[12] Zhu AX, Kudo M, Assenat E, Cattan S, Kang YK, Lim HY (2014). Effect of everolimus on survival in advanced hepatocellular carcinoma after failure of sorafenib: the EVOLVE-1 randomized clinical trial. JAMA.

[13] Llovet JM, Decaens T, Raoul JL, Boucher E, Kudo M, Chang C (2013). Brivanib in patients with advanced hepatocellular carcinoma who were intolerant to sorafenib or for whom sorafenib failed: results from the randomized phase III BRISK-PS study. J Clin Oncol.

[14] Dufour JF, Bargellini I, De Maria N, De Simone P, Goulis I, Marinho RT (2013). Intermediate hepatocellular carcinoma: current treatments and future perspectives. Ann Oncol.

[15] Raoul JL, Sangro B, Forner A, Mazzaferro V, Piscaglia F, Bolondi L (2011). Evolving strategies for the management of intermediate-stage hepatocellular carcinoma: available evidence and expert opinion on the use of transarterial chemoembolization. Cancer Treat Rev.

[16] Hucke F, Sieghart W, Pinter M, Graziadei I, Vogel W, Muller C (2014). The ART-strategy: sequential assessment of the ART score predicts outcome of patients with hepatocellular carcinoma re-treated with TACE. J Hepatol.

[17] Sieghart W, Hucke F, Pinter M, Graziadei I, Vogel W, Muller C (2013). The ART of decision making: retreatment with transarterial chemoembolization in patients with hepatocellular carcinoma. Hepatology.

[18] Johnson GE, Monsky WL, Valji K, Hippe DS, Padia SA (2016). Yttrium-90 radioembolization as a salvage treatment following chemoembolization for hepatocellular carcinoma. J Vasc Interv Radiol.

[19] Salem R, Gordon AC, Mouli S, Hickey R, Kallini J, Gabr A, et al. Y90 radioembolization significantly prolongs time to progression compared with chemoembolization in patients with hepatocellular carcinoma. Gastroenterology. 2016; 10.1053/j.gastro.2016.08.029. 10.1053/j.gastro.2016.08.029 PMC5124387 27575820

[20] Vilgrain V, Pereira H, Assenat E, Guiu B, Ilonca AD, Pageaux GP, et al. Efficacy and safety of selective internal radiotherapy with yttrium-90 resin microspheres compared with sorafenib in locally advanced and inoperable hepatocellular carcinoma (SARAH): an open-label randomised controlled phase 3 trial. Lancet Oncol. 2017; 10.1016/S1470-2045(17)30683-6. 10.1016/S1470-2045(17)30683-6 29107679

[21] Chow PHW, Gandhi M (2017). Asia-Pacific Hepatocellular Carcinoma Trials Group. Phase III multi-centre open-label randomized controlled trial of selective internal radiation therapy (SIRT) versus sorafenib in locally advanced hepatocellular carcinoma: The SIRveNIB study. J Clin Oncol.

[22] Lau WY, Leung WT, Ho S, Leung NW, Chan M, Lin J (1994). Treatment of inoperable hepatocellular carcinoma with intrahepatic arterial yttrium-90 microspheres: a phase I and II study. Br J Cancer.

[23] Garin E, Rolland Y, Pracht M, Le Sourd S, Laffont S, Mesbah H (2017). High impact of macroaggregated albumin-based tumour dose on response and overall survival in hepatocellular carcinoma patients treated with (90) Y-loaded glass microsphere radioembolization. Liver Int.

[24] Bruix J, Raoul JL, Sherman M, Mazzaferro V, Bolondi L, Craxi A (2012). Efficacy and safety of sorafenib in patients with advanced hepatocellular carcinoma: subanalyses of a phase III trial. J Hepatol.

